# Antioxidant Activity of Brazilian Vegetables and Its Relation with Phenolic Composition

**DOI:** 10.3390/ijms13078943

**Published:** 2012-07-18

**Authors:** Ana P. Tiveron, Priscilla S. Melo, Keityane B. Bergamaschi, Thais M. F. S. Vieira, Marisa A. B. Regitano-d’Arce, Severino M. Alencar

**Affiliations:** Department of Agri-Food Industry, Food and Nutrition, “Luiz de Queiroz” College of Agriculture, University of São Paulo, Piracicaba, SP, Av. Pádua Dias, 11, CEP 13418-900, Brazil; E-Mails: anapaulativeron@gmail.com (A.P.T.); priscilla_esalq@yahoo.com.br (P.S.M.); keityberga@yahoo.com.br (K.B.B.); tvieira@usp.br (T.M.F.S.V.); marisadarce@usp.br (M.A.B.R.A.)

**Keywords:** antioxidant activity, phenolic compounds, vegetables, GC-MS

## Abstract

Vegetables are widely consumed in Brazil and exported to several countries. This study was performed to evaluate the phenolic content and antioxidant activity of vegetables commonly consumed in Brazil using five different methods, namely DPPH and ABTS free radical, β-carotene bleaching, reduction of Fe^3+^ (FRAP), oxidative stability in Rancimat, and the chemical composition using gas chromatography-mass spectrometry (GC-MS). The content of phenolic compounds ranged from 1.2 mg GA/g (carrot) to 16.9 mg GA/g (lettuce). Vegetables presenting the highest antioxidant activity were lettuce (77.2 μmol Trolox/g DPPH^•^; 447.1 μmol F^2+^/g FRAP), turmeric (118.6 μmol Trolox/g ABTS^•+^; 92.8% β-carotene), watercress and broccoli (protective factor 1.29—Rancimat method). Artichoke, spinach, broccoli, and asparagus also showed considerable antioxidant activity. The most frequent phenolic compounds identified by GC-MS were ferulic, caffeic, *p-*coumaric, 2-dihydroxybenzoic, 2,5-dihydroxybenzoic acids, and quercetin. We observed antioxidant activity in several vegetables and our results point out their importance in the diet.

## 1. Introduction

Vegetables are widely produced in Brazil both for local consumption and export to several countries. Brazil is a country that presents great agro-ecological diversity and a large production of vegetables. Recent studies have shown the importance of vegetables in a healthy diet and to prevent degenerative diseases caused by oxidative stress [1]. Vitamins and phytochemicals, such as ascorbic acid, carotenoids, polyphenols, and fiber have been regarded as the bioactive substances responsible for these effects [2].

Phenolic compounds are secondary metabolites commonly found in plants, useful in the defensive function against pathogens and radiation, and are directly involved in the antioxidant activity [3–5].

Antioxidants can be defined as any substance that, present in low concentration compared to an oxidized substrate, effectively delays or inhibits oxidation of the substrate [6]. For the food industry, it is also highly interesting to find new and safe antioxidants from natural sources. Although synthetic antioxidants are very effective and stable, they have limited use in many countries due to the possibility of causing adverse effects on human health [7,8].

Several techniques have been used to determine the *in vitro* antioxidant activity in order to allow rapid screening of promising substances and/or mixtures. In the last few years, evaluation of the antioxidant potential of food, as well as natural, pharmaceutical, and cosmetic products has been increasing. The interest on this field began to expand in the 1990s based on the observation that many natural products have beneficial effects on human health. Furthermore, the research and validation of methodologies for evaluating the antioxidant activity in complex matrices such as food, natural products, and biological fluids have also been improved [5,9,10]. Although many methods have been developed and tested in the literature, their advantages and limitations are still under discussion and no consensus has been reached to define a unique standard method capable of encompassing all the peculiarities exhibited by the different classes of antioxidants [11]. Many methods are available for analyzing antioxidant activity, with different concepts, mechanisms of action, ways of expressing results, and applications [12–14]. On the one hand, indirect methods, involving electron transfer reactions, such as ABTS^•+^, DPPH^•^, and FRAP, are easier to apply, but present some limitations. In this case, the methods evaluate the free radical scavenging ability of antioxidant compounds, and this does not necessarily correspond to the real oxidative degradation, although, in some circumstances, the donation of hydrogen atoms (or electrons) correlates with the antioxidant activity. On the other hand, according to Becker *et al.* [15], direct methods, such as β-carotene and Rancimat, are characterized by their ability to inhibit or halt lipid oxidation in model systems, based on measuring changes in the concentration of compounds being oxidized, on oxygen depletion, or on formation of oxidation products. Therefore, since these chemically distinct methods are based on different reaction mechanisms, it is important to use different methods in order to obtain a more thorough assessment of the antioxidant potential of a sample [10,16]. Several analytical methods have been used to evaluate antioxidant activity by free radical scavenging, but besides being one of the fastest, ABTS^•+^ method also provides good solubility, which allows the analyses of both lipophilic and hydrophilic compounds [17].

This study evaluates the antioxidant activity properties of the main vegetables produced and consumed in Brazil. Antioxidant properties are determined in various test systems with different mechanisms of action, including antioxidant activity by scavenging abilities on DPPH (2,2-diphenyl-1-picrylhydrazyl radical) and ABTS (2,2′-azinobis-3-ethylbenzotiazoline-6-sulfonic acid radical), reducing power test, β-carotene bleaching and oxidative stability by the Rancimat method. Content and profile of potential antioxidant in these vegetables are also examined.

## 2. Results and Discussion

Phenolic compounds are responsible for the antioxidant activity of vegetables. Although in recent years the antioxidant analysis of vegetables has been extensively researched worldwide [18–22], very few studies have been carried out to assess vegetables grown in Brazil and consumed locally or exported to several countries.

**Figure 1:**
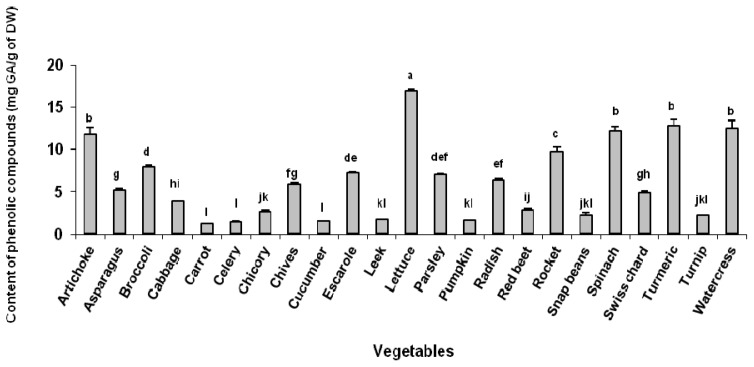
Content of phenolic compounds (mg GA/g of DW) in several vegetables grown in Brazil; means followed by different letters differ statistically (*p* < 0.05) by Tukey test.

The content of phenolic compounds found in our samples ranged from 1.2 to 16.9 mg GA/g sample (dry weight—DW), in carrot and lettuce, respectively (Figure 1). These results were different from the findings reported by Chu *et al.* [23], since in their study lettuce was one of the vegetables that presented the lowest total phenolic content, using the same method. Llorach *et al*. [22] found higher levels of phenolic compounds in red-leafed varieties of lettuce compared to green ones. According to Gobbo-Neto and Lopes [24], several factors such as seasonality, temperature, water availability, UV radiation, soil nutrients, pollution, and pathogen attack can affect the content of secondary metabolites in vegetables, such as phenolic compounds.

According to the literature, phenolic compounds found in plants have antioxidant and anticancer activities. In this regard, some authors highlighted the antioxidant activity of curcumin, a naturally occurring phenolic compound of turmeric that is responsible for its peculiar color and important antioxidant and anticancer activities [25–27].

The results presented in Table 1 show that using the ABTS^•+^ method, turmeric had the highest antioxidant activity (118.6 μmol Trolox/g), followed by watercress (97.1 μmol Trolox/g) and lettuce (85.8 μmol Trolox/g).

The analysis of the antioxidant activity of commonly consumed vegetables grown in Colorado (USA) using the ABTS^•+^ method showed that spinach and broccoli presented antioxidant activity of approximately 50 μmol Trolox/g and 40 μmol Trolox/g, respectively, a result similar to our findings, of 41.2 μmol Trolox/g and 43.0 μmol Trolox/g, respectively [20].

Turmeric (92.8%) and lettuce (90%) showed the highest antioxidant activity by the β-carotene bleaching method, while turnip (3.4%) presented the lowest result. One factor that may influence this analysis is the medium where the reaction occurs, characterized as an emulsion, with polar and non-polar regions simultaneously. Therefore, depending on the polarity of the sample, it can interact more or less intensely with the emulsion.

The highest ability to reduce Fe^3+^ to Fe^2+^ was found in lettuce (447.1 μmol Fe^2+^/g), watercress (277.4 μmol Fe^2+^/g), and spinach (273.3 μmol Fe^2+^/g) (Table 1). Studying wild artichoke from Slovakia, Kukić *et al*. [28] reported 350 μmol Fe^2+^/g in dry basis for ethanol extracts, and 340 μmol Fe^2+^/g for aqueous extracts. In contrast, in the present study, Brazilian artichoke showed much lower ability to reduce Fe^3+^ (98.7 μmol Fe^2+^/g).

The Rancimat method is widely used for the determination of the oxidative stability of natural fats and oils. The induction period is characterized by change in conductivity of deionized water due to oxidation-generated products. The process is carried out under high temperatures and constant aeration. All vegetables tested, except the escarole, had a longer induction period compared to the control (pure soybean oil, without added antioxidants), with a protection factor and consequent antioxidant activity (Table 1). The vegetables that showed the highest protection factors were watercress (1.29), broccoli (1.29) and chives (1.24), while escarole showed no protection against lipid oxidation. It could be inferred that, except for escarole, soybean oil added to vegetable extracts required a longer time to form free radicals, reactive molecules that trigger the initiation phase of the oxidation process, thus delaying the propagation phase and, consequently, the termination phase.

The highest antioxidant activities by the DPPH^•^ method were found in lettuce, artichoke, turmeric, spinach, escarole, and watercress (77.2, 70.1, 57.6, 50.9, 48.1, and 44.0 μmol Trolox/g, respectively) (Table 1).

**Figure 2:**
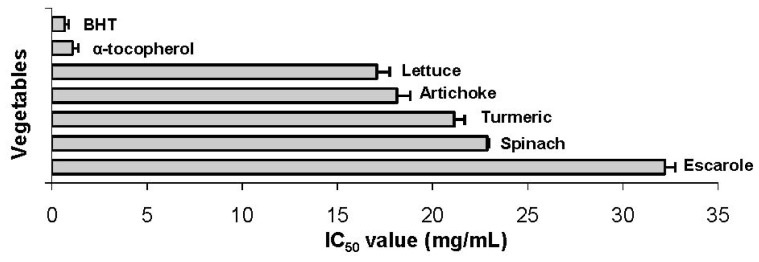
Concentration of vegetable extract required to reduce the initial DPPH radical by 50%.

Based on the highest results using the DPPH^•^ method, the IC_50_ of lettuce, artichoke, turmeric, spinach, and escarole were calculated and are shown in Figure 2. The vegetable that showed the lowest extract concentration to reduce the initial amount of DPPH radical by 50% was lettuce (17.07 mg/mL), followed by artichoke (18.14 mg/mL), turmeric (21.14 mg/mL), spinach (22.87 mg/mL), and escarole (32.2 mg/mL). Vegetables presenting the lowest IC_50_ values can be considered better in terms of antioxidant activity, since a lower concentration to reduce the DPPH free radical by 50% is required.

All vegetables except for escarole have considerable amounts of total phenolic compounds and consequent significant antioxidant activity. The correlation between them becomes important and is presented in Table 2.

The amount of phenolic compounds found in the vegetables evaluated in this study showed no direct relationship with their antioxidant activity using some methods. The lowest correlation between phenolic compound content and antioxidant activity was observed for the Rancimat method (*r*^2^ = 3.47%), whereas the DPPH^•^ and FRAP methods (80.64% and 68.60%, respectively) showed the highest correlations.

Table 3 shows the correlation between the methodologies used in this study to evaluate antioxidant activity in vegetables. The results reveal lack of consistency among these methodologies. This fact could be explained by the different characteristics and mechanisms of action of the bioactive compounds present in the samples as well as the different principles used to detect antioxidant properties in each method.

The best correlations were found for DPPH^•^ *vs*. FRAP (56.65%) and ABTS^•+^ *vs*. FRAP (56.08%), while the Rancimat *vs*. FRAP did not show a good correlation (0.09%). The method that best correlated with Rancimat was β-caroten (19.9%), both considered direct methods.

Therefore, despite the difficulty to choose the most appropriate method for the evaluation of antioxidant activity, the most commonly accepted, validated, and standardized methods, with more information available should be chosen.

The results of the phenolic composition obtained using GC-MS are presented in Table 4. Several compounds derived from benzoic acid (3-hydroxybenzoic, syringic, 2,5-dihydroxybenzoic, 2-hydroxybenzoic, gallic, and 2,4-dihydroxybenzoic acids) and from cinnamic acid (sinapic, *p*-coumaric, and caffeic acids) were found. The most abundant phenolic compound derived from cinnamic acid present in the samples assessed in this study was caffeic acid, and spinach (53.54%) showed the greatest amount of this compound.

Regarding flavonoids, quercetin was found in lettuce (2.02%), chicory (1.32%), asparagus (5.56%) chives (0.93%) and snap beans (8.67%) and kaempferol in chives (4.60%), snap beans (3.41%), and escarole (3.19%), already known for its high antioxidant activity (Table 4). Llorach *et al*. [22] detected luteolin and quercetin derivatives in lettuce, in amounts that varied according to the cultivar. DuPont *et al*. [29] analyzed the flavonoids of *Cichorium endivia* in the United Kingdom, and also identified the presence of kaempferol, corroborating the results found in this work.

The ascorbic acid identified in some of our samples may have contributed to the antioxidant activity of vegetables, such as in the cases of broccoli, cabbage, chives, parsley, rocket, and watercress, since this is a compound with high electron donation capacity.

## 3. Experimental Section

### 3.1. Chemicals and Reagents

Gallic acid, 2,2-diphenyl-1-picryl-hydrazine (DPPH^•^), 2,2′-azinobis-3-ethylbenzotiazoline-6-sulfonic acid (ABTS^•+^), β-carotene, linoleic acid, ferric chloride, ferrous sulfate, tripyridyltriazine, *N*-methyl-*N*-(trimethylsilyl)trifluoroacetamide (MSTFA), and standards for GC-MS were purchased from Sigma-Aldrich (St. Louis, MO, USA). The other reagents were purchased from local sources. The cartridges used for SPE-LC18 chromatographic analysis were obtained from Supelco (Bellefonte, PA, USA).

### 3.2. Sample Collection and Extraction

All the vegetables used were produced in the São Paulo State, in Southeastern Brazil, wet tropical climate, 22°43′31″ S and 47°38′57″ W, from January to October. The following vegetable species were assessed: (1) artichoke (*Cynara scolymus* L.); (2) asparagus (*Asparagus officinalis* L.); (3) broccoli (*Brassica oleracea* L. var. *italic* Plenck); (4) cabbage (*Brassica oleracea* L. var. *capitata*); (5) carrot (*Daucus carota* L.); (6) celery (*Apium graveolens* L.); (7) chicory (*Cichorium intybus* L.); (8) chives (*Allium fistolosum* L.); (9) cucumber (*Cucumis sativus* L.); (10) escarole (*Cichorium endivia* L.); (11) leek (*Allium porrum* L.); (12) lettuce (*Lactuca sativa* L.); (13) parsley (*Petroselinum crispum* (Mill.) Nym.); (14) pumpkin (*Cucurbita maxima* Duch.); (15) radish (*Raphanus sativus* L.); (16) red beet (*Beta vulgaris* L.); (17) rocket (*Eruca sativa* L.); (18) snap beans (*Phaseolus vulgaris* L.); (19) spinach (*Tetragonia expansa* L.); (20) Swiss chard (*Beta vulgaris* L. var. *cicla*); (21) turmeric (*Curcuma longa* L.); (22) turnip (*Brassica rapa* L.); (23) watercress (*Nasturtium officinale*).

The extraction of the compounds of interest from the vegetable samples was performed according to the method described by Kähkönen *et al*. [3] with some modifications. Samples were first frozen at −18 °C and then lyophilised. After that, the material was ground and 1 g of each vegetable powder was placed in Falcon tubes, mixed with 20 mL of 80% ethanol (v/v), and shaken thoroughly. Subsequently, the tubes were sonicated for 5 min, centrifuged for 15 min at 5000× g, and the supernatant collected for analysis. All samples were extracted in triplicate.

### 3.3. Determination of Total Phenolic Content

Total phenolic content analysis was performed using the Folin Ciocalteau spectrophotometric method described by Singleton *et al*. [30,31]. The vegetable extracts were diluted in ethanol 80% and 0.5 mL of the solution obtained was transferred to a tube with 2.5 mL of Folin Ciocalteau reagent diluted in water at 1:10. The mixture was allowed to sit for 3–8 min, then 2 mL of sodium carbonate 4% was added, and the tubes were kept in the dark for 2 h. Afterwards, the absorbance was measured at 740 nm using a UV-mini 1240 spectrophotometer (Shimadzu, Japan). A blank test was also performed under the same conditions and the results of total phenolic compounds were expressed as gallic acid equivalent (mg GA/g sample DW), based on a calibration curve of gallic acid in the concentration range of 5 to 80 μg/mL.

### 3.4. DPPH Free Radical Scavenging Assay and IC_50_

The reaction mixture consisted of 0.5 mL of standards or vegetable extracts, 3.0 mL of pure ethanol, and 0.3 mL of DPPH radical in ethanol solution 0.5 mM, which was incubated at room temperature for 45 min, and the activity was expressed in μmol Trolox/g of sample DW [32]. The calibration curve was constructed with the standard Trolox in the concentration range of 0 to 200 μM Trolox.

Several vegetable extract concentrations were used, and readings were monitored at 517 nm, using a UV-mini 1240 spectrophotometer (Shimadzu, Japan), every 20 min for 140 or 160 min, depending on the sample, until a constant reading was obtained. The antioxidant activity measured by DPPH free radical method can be expressed as IC_50_, *i.e.*, the antioxidant concentration required to reduce the initial DPPH radical by 50%. The concentration of vegetable samples required to reduce the initial DPPH radical by 50% is expressed in mg/mL.

### 3.5. Antioxidant Activity Using β-Carotene Bleaching Method

The antioxidant activity assessment was performed according to the method described by Emmons *et al*. [33] with some modifications. An emulsion was prepared by dissolving 10 mg of β-carotene in 100 mL of chloroform PA and aliquots of 3 mL were added to 40 mg of linoleic acid and 400 mg of Tween 40. The chloroform was removed using a stream of nitrogen and the residue obtained was redissolved in 100 mL of water aerated for 30 min. Aliquots of 3 mL of the resultant β-carotene/linoleic acid emulsion were mixed with 50 μL of the vegetable extracts and incubated in a water bath at 50 °C. The emulsion oxidation was monitored using a UV-mini 1240 spectrophotometer (Shimadzu, Japan) at 470 nm, at baseline and at 20-min intervals for 2 h. Control samples contained only solvent in place of the vegetable extracts. BHT and α-tocopherol (200 ppm) were used as reference standards. The antioxidant activity was expressed as percentage of relative inhibition compared to control samples after 120 min and calculated as:

%AA=[(DRc-DRs)/DRc]×100

where AA = antioxidant activity; DRc = control degradation rate (ln (a/b)/120); DRs = degradation rate in the presence of standard or vegetable extract (ln (a/b)/120); a = absorbance at initial time (0 min); b = absorbance at final time (120 min).

### 3.6. Antioxidant Activity Using ABTS^•+^ Assay

The antioxidant activity by the ABTS^•+^ method (2,2′-azinobis-3-ethylbenzotiazoline-6-sulfonic acid) was assessed according to the method described by Re *et al.* [17] with modifications. The ABTS radical was formed through the reaction of ABTS^•+^ solution 7 mM with potassium persulfate solution 140 mM, incubated at 25 °C in the dark for 12–16 h. Once formed, the radical was diluted with ethanol P.A. to an absorbance of 0.700 ± 0.020 at 734 nm. Three different dilutions of each vegetable extract were prepared in triplicate. After that, 30 μL of each vegetable extract dilution were transferred to test tubes with 3.0 mL of ABTS radical in the dark. The absorbance was read at 734 nm after 6 min of the reaction using ethanol as a blank. Trolox, a synthetic water-soluble antioxidant analogue of vitamin E, was used as reference at concentrations ranging from 100 to 2000 μM and the results were expressed as μM Trolox/g sample.

### 3.7. Ferric Reducing Antioxidant Power Assay (FRAP)

To determine the antioxidant activity by iron reduction, using the ferric reducing antioxidant power (FRAP) assay, we followed the methodology described by Benzie and Strain [34] with some modifications. FRAP measures the ferric reducing ability of the samples, in acidic medium (pH 3.6), forming an intense blue color as the ferric tripyridyltriazine (Fe^3+^-TPTZ) complex is reduced to the ferrous (Fe^2+^) form. FRAP reagent was prepared immediately before analysis by mixing 25 mL of acetate buffer (300 mM, pH 3.6), 2.5 mL of TPTZ solution (10 mM TPTZ in 40 mM HCl), and 2.5 mL of FeCl_3_ (20 mM) in aqueous solution. An aliquot of 100 μL of the vegetable extracts was added to 3 mL of FRAP reagent and incubated in a water bath at 37 °C for 30 min. After this time, the absorbance was measured using a UV-mini 1240 spectrophotometer (Shimadzu, Japan) that was reset with FRAP solution. The calibration curve was constructed using ferrous sulfate (100–2000 μM) and the results were expressed in μmol Fe^2+^/mg.

### 3.8. Oxidative Stability—Rancimat

Samples of 5 g of pure soybean oil (supplied by Cargill, without added antioxidants) were mixed with the vegetable extracts at a concentration of 100 ppm, based on the content of phenolic compounds. The oxidative stability index of this mixture was measured by the Rancimat method according to AOCS Cd 12b-92 [35] at 110 ± 1 °C and a flow rate of 9 L/h of dry air, using 743 Rancimat (Metrohm AG, CH-9100 Herisau, Switzerland). The conductivity increase due to the accumulation of oxidized compounds plotted as a function of the reaction time allowed the construction of the curve and the calculation of the induction period (IP). A control was prepared with pure soybean oil without added antioxidants and samples containing synthetic antioxidant BHT (100 ppm) were also submitted to this analysis.

The protection factor was calculated as:

%PF=(PIa/PIc)×100

where PF = protection factor; PIa = IP of the oil with the vegetable extracts or standards; Plc = IP of the control (oil without the vegetable extracts or standards).

### 3.9. Chromatographic Analysis

#### 3.9.1. Removal of Outliers from Samples Using the Solid Phase Extraction (SPE) Technique

The Solid Phase Extraction (SPE) technique was employed for the removal of sugars, which could mask the compounds of interest. This technique has been increasingly used because it is quick, efficient, and requires very small volumes of samples and solvents. LC-18 SPE cartridges (2 g, Supelco, Bellefonte, PA, USA) were conditioned with methanol and acidic water (pH = 2.0). Subsequently, 4 mL of each vegetable ethanol extract once again evaporated and redissolved in 4 mL of water were added to their respective cartridges. After the vegetable extract completely passed through, the column was washed with sufficient acidic water to remove the sugars. Compounds of interest were eluted with methanol into coded glass vials and their bands were identified under UV light.

#### 3.9.2. Derivatization—Formation of Trimethylsilyl Derivatives (TMS)

Prior to the GC-MS analysis, the samples were submitted to a crucial stage called derivatization. GC-MS is only useful for the analysis of gases, volatile, and thermally stable substances. Samples not showing this profile, presenting high molecular weight compounds and/or strongly polar functional groups, require a derivatization procedure, a reaction which transforms a substance of interest into a product of similar chemical structure, called a derivative, presenting characteristics suitable for analysis [36]. Chemical derivatization is widely used to reduce the polarity of functional groups and facilitate their separation during GC-MS analysis.

The fractions obtained after purification were added to 100 μL of derivatizing reagent MSTFA. The reaction mixture was homogenized and incubated at 70 °C for 10 min. The reagent was evaporated under a stream of nitrogen and trimethylsilyl (TMS) derivatives were rediluted in hexane (800 μL). After homogenization, the supernatant was transferred to a vial and injected into the GC-MS system.

#### 3.9.3. Gas Chromatography-Mass Spectrometry (GC-MS)

GC-MS analyses of the vegetable extracts were performed on a gas chromatograph GC 2010 (Shimadzu Corp., Kyoto, Japan) coupled to a mass spectrometer QP 2010 Plus (Shimadzu Corp., Kyoto, Japan). Derivatized samples were separated using a capillary column (RTX-5MS 30 m × 0.25 mm × 0.25 μm). The temperature program started at 80 °C (1 min), increasing at 20 °C/min to 250 °C, remaining at 250 °C for 1 min (9 min 30 s), increasing at 6 °C/min to 300 °C, remaining at 300 °C for 5 min (13 min 20 s), increasing at 15 °C/min to 310 °C, remaining at 310 °C for 5 min (5 min 40 s), increasing at 20 °C/min to the final temperature of 320 °C, remaining at 320 °C for 10 min (10 min 30 s), totaling 40 min of analysis. Helium was used as the carrier gas, the injector temperature was 280 °C, and the injection volume was 0.5 μL in splitless mode. The interface was maintained at 280 °C and the detector was operated in the scanning mode (*m/z* 40–800). Data integration was performed using the LabSolutions-GCMS software. Flavonoids, phenolic acids, and derivatives were identified by comparing their retention time and ion fragmentation with coded and authentic standards (quercetin, apigenin, kaempferol, kaempferide, rutin, epicatechin, catechin, resveratrol, ferulic acid, caffeic acid, *p*-coumaric acid, cinnamic acid) eluted under the same conditions as well as with the Wiley Version 8 library.

### 3.10. Statistical Analysis

The results obtained were statistically analyzed using the SAS software [37] and the analysis of variance using the general linear models (GLM) procedure. The Tukey test at 5% probability was used for mean comparison.

## 4. Conclusions

The amount of phenolic compounds present in the vegetables analyzed showed no direct relationship with the antioxidant activity in a large number of samples. Based on this finding, it is possible to affirm that different phenolic compounds and some non-phenolic substances present different antioxidant activities, and that the presence of high levels of certain classes of compounds in vegetables does not always guarantee higher antioxidant power. The highest correlation of the amount of phenolic compounds was found using the DPPH^•^ method. Lettuce, turmeric, watercress, and broccoli presented the highest antioxidant activity among the Brazilian vegetables analyzed.

The samples did not present the same results in all methodologies and no defined order of antioxidant activity could be found. One possible explanation for this could be the difference in the chemical composition of vegetables and different mediums and principles of analyses. Due to the great chemical diversity of the compounds found in this study, the use of more than one method is more suitable for the analysis of *in vitro* antioxidant activity of these vegetables.

Several phenolic compounds with high antioxidant activity could be identified using GC-MS in vegetables, mainly phenolic acids, some of them present in high concentrations, such as caffeic acid in spinach, chicory, and escarole.

## Figures and Tables

**Table 1:** Antioxidant activity of commonly consumed vegetables in Brazil.

Vegetable	Antioxidant activity				

	ABTS^•+^ (μmol Trolox/g DW)	β-carotene (%)	FRAP (μmol Fe^2+^/g DW)	Rancimat (protection factor)	DPPH (μmol Trolox/g DW)
Artichoke	39.9 ± 3.54 ^defg^	57.7 ± 1.3 ^fgh^	98.7 ± 6.10 ^h^	1.09 ± 0.005 ^cde^	70.1 ± 5.44 ^b^
Asparagus	37.5 ± 0.80 ^efg^	88.3 ± 2.8 ^abc^	95.2 ± 2.11 ^h^	1.11 ± 0.04 ^cd^	15.8 ± 0.38 ^h^
Broccoli	43.0 ± 0.48 ^def^	54.7 ± 2.7 ^gh^	188.0 ± 3.64 ^d^	1.29 ± 0.05 ^a^	33.4 ± 0.97 ^f^
Cabbage	15.3 ± 1.58 ^ijk^	16.7 ± 4.7 ^jk^	49.7 ± 2.32 ^i^	*	8.6 ± 0.35 ^ijk^
Carrot	0.9 ± 1.13 ^o^	49.5 ± 7.5 ^h^	13.2 ± 0.92 ^k^	*	3.5 ± 0.07 ^mn^
Celery	2.9 ± 1.38 ^lmno^	36.2 ± 2.2 ^i^	13.7 ± 0.67 ^k^	*	3.8 ± 0.33 ^lmn^
Chicory	54.9 ± 4.53 ^de^	82.2 ± 4.9 ^c^	217. 2 ± 6.85 ^c^	*	9.9 ± 0.93 ^ijk^
Chives	25.8 ± 1.06 ^fghi^	28.8 ± 4.8 ^i^	130.8 ± 3.58 ^fg^	1.24 ± 0.03 ^ab^	8.2 ± 0.14 ^ijkl^
Cucumber	10.1 ± 1.48 ^jklm^	67.6 ± 3.0 ^de^	16.3 ± 1.64 ^jk^	*	2.3 ± 0.23 ^n^
Escarole	19.1 ± 5.22 ^hij^	82.0 ± 1.9 ^c^	148.5 ±3.21 ^ef^	0.98 ± 0.01 ^f^	48.1 ± 0.61 ^de^
Leek	8.3 ± 0.21 ^jklmn^	9.4 ± 0.6 ^lm^	12.2 ± 1.06 ^k^	*	3.2 ± 0.12 ^mn^
Lettuce	85.8 ± 5.34 ^bc^	90.0 ± 3.0 ^ab^	447.1 ± 4.55 ^a^	1.02 ± 0.002 ^ef^	77.2 ± 0.83 ^a^
Parsley	30.7 ± 3.98^fgh^	60.7 ± 2.1 ^efg^	104.8 ± 1.44 ^gh^	1.05 ± 0.01 ^def^	10.8 ± 0.47 ^ij^
Pumpkin	11.0 ± 1.39 ^jkl^	54.3 ± 5.3 ^gh^	19.5 ± 1.77 ^jk^	*	5.8 ± 0.095 ^klmn^
Radish	61.7 ± 0.21 ^cd^	22.2 ± 6.0 ^j^	90.0 ± 4.39 ^h^	1.17 ± 0.01 ^bc^	26.1 ± 0.40 ^g^
Red beet	2.5 ± 2.03 ^no^	15.5 ± 9.7 ^jk^	43.3 ±1.07 ^ij^	*	11.5 ± 0.13 ^hij^
Rocket	25.5 ± 0.75 ^ghi^	54.0 ± 4.2 ^gh^	113.8± 5.94 ^gh^	1.04 ± 0.05 ^def^	11.6 ± 0.26 ^hi^
Snap beans	3.4 ± 0.51 ^lmno^	67.0 ± 2.3 ^de^	15.7 ± 1.26 ^jk^	*	3.8 ± 0.26 ^lmn^
Spinach	41.2 ± 5.42 ^defg^	85.6 ± 1.5 ^bc^	273.3 ± 6.10 ^b^	1.16 ± 0.05 ^bc^	50.9 ± 0.31 ^d^
Swiss chard	25.4 ± 2.12 ^ghi^	65.6 ± 3.9 ^def^	50.5 ± 4.36 ^i^	1.10 ± 0.01 ^cde^	9.1 ± 0.12 ^ijk^
Turmeric	118.6 ± 3.77 ^a^	92.8 ± 1.3 ^a^	169.1 ± 4.0 ^de^	1.04 ± 0.04 ^def^	57.6 ± 2.39 ^c^
Turnip	6.5 ± 0.42 ^klmn^	3.4 ± 1.7 ^m^	15.5 ±0.43 ^jk^	*	7.1 ± 0.33 ^jklm^
Watercress	97.1 ± 2.82 ^ab^	70.9 ± 4.6 ^d^	277.4 ± 8.78 ^b^	1.29 ± 0.01 ^a^	44.0 ± 2.53 ^e^

Reference values: BHT (100 ppm)—1666.1 ± 6.0 μmol Trolox/g (ABTS^•+^); 93.9 ± 0.21% (β-carotene); 11762.0 ± 1.5 μmol Trolox/g (FRAP); 1.10 ± 0.090 (Rancimat); 200.4 ± 0.3 μmol Trolox/g (DPPH); α-tocopherol (100 ppm)—2855.2 ± 1.9 μmol Trolox/g (ABTS^•+^); 88 ± 1.6% (β-carotene); 3462.9 ± 3.4 μmol Trolox/g (FRAP); 1507.0 ± 1.2 μmol Trolox/g (DPPH); Values represent the average of triplicates ± standard deviation; means followed by different letters in the same column differ statistically (*p* < 0.05) by the Tukey test.

* Induction period not calculated due to the large amount of samples required to achieve the concentration of 100 ppm of total phenolic compounds;

ABTS^•+^: 2,2′-azinobis-3-ethylbenzotiazoline-6-sulfonic acid; FRAP: ferric reducing antioxidant power; DPPH: 2,2-diphenyl-1-picryl-hydrazine.

**Table 2:** Pearson’s correlation coefficient between total phenolic compounds (TPC) and total antioxidant activity.

Correlation	*r*	*r*^2^ (%)
TPC *vs*. DPPH	0.89	80.64
TPC *vs*. β-carotene	0.52	27.49
TPC *vs*. ABTS	0.79	61.85
TPC *vs*. FRAP	0.82	68.60
TPC *vs*. Rancimat	0.18	3.47

TPC: total phenolic content; DPPH: 2,2-diphenyl-1-picryl-hydrazine; ABTS: 2,2′-azinobis-3-ethylbenzotiazoline-6-sulfonic acid; FRAP: ferric reducing antioxidant power.

**Table 3:** Pearson’s correlation coefficient between the different antioxidant activity methodologies.

Correlation	*r*	*r*^2^ (%)
DPPH *vs*. β-carotene	0.52	27.36
DPPH *vs*. ABTS	0.71	51.03
DPPH *vs*. FRAP	0.75	56.65
DPPH *vs*. Rancimat	0.22	4.93
β-carotene *vs*. ABTS	0.53	28.67
β-carotene *vs*. FRAP	0.58	34.07
β-carotene *vs*. Rancimat	0.44	19.9
ABTS *vs*. FRAP	0.74	56.08
ABTS *vs*. Rancimat	0.09	0.97
FRAP *vs*. Rancimat	0.03	0.09

DPPH: 2,2-diphenyl-1-picryl-hydrazine; ABTS: 2,2′-azinobis-3-ethylbenzotiazoline-6-sulfonic acid; FRAP: ferric reducing antioxidant power.

**Table 4:** Concentration (%) of compounds identified in ethanol extracts of the vegetables analyzed using GC-MS.

Vegetable ethanol extract	Area of the component (%)													

	1	2	3	4	5	6	7	8	9	10	11	12	13	14
Artichoke	–	–	–	0.01	–	–	–	–	–	32.46	–	–	–	–
Asparagus	–	–	–	–	0.15	–	–	–	2.61	1.40	–	5.56	–	–
Broccoli	1.12	–	–	–	0.51	–	6.72	–	1.95	0.31	–	–	–	–
Cabbage	0.63	–	–	–	–	–	12.61	0.41	12.61	0.28	–	–	–	–
Carrot	–	0.13	–	–	–	–	–	0.42	–	0.49	–	–	0.78	–
Celery	–	–	–	1.68	–	–	–	–	1.08	9.88	–	–	–	0.99
Chicory	–	–	–	–	–	–	–	0.39	–	47.04	–	1.32	–	–
Chives	0.26	–	–	–	–	–	0.10	2.72	15.60	0.56	4.60	0.93	0.12	–
Cucumber	–	–	–	1.43	14.23	–	–	–	0.22	–	–	–	–	–
Escarole	–	–	–	–	–	–	–	–	0.22	42.53	3.19	–	–	–
Leek	–	–	–	–	–	–	–	0.19	0.96	–	–	–	–	–
Lettuce	–	–	–	–	–	–	–	0.60	–	26.92	–	2.02	–	0.16
Parsley	1.08	–	–	0.06	0.82	–	–	–	–	0.14	–	–	–	0.41
Pumpkin	–	–	–	–	–	0.32	–	–	–	–	–	–	–	–
Radish	–	0.89	–	–	–	–	–	1.81	1.81	1.18	–	–	–	–
Red beet	–	–	9.54	0.52	–	–	–	0.43	0.82	–	–	–	–	–
Rocket	5.31	–	–	0.14	–	–	0.21	–	–	–	–	–	–	–
Snap beans	–	–	–	–	1.14	–	–	2.63	0.71	0.50	3.41	8.67	9.73	1.67
Spinach	–	–	–	–	–	–	–	–	1.60	53.54	–	–	–	–
Swiss chard	–	–	–	–	0.24	–	10.85	–	1.38	–	–	–	–	–
Turmeric	–	–	–	–	–	–	–	–	–	–	–	–	0.57	0.35
Turnip	–	0.96	–	–	–	–	–	0.42	0.78	–	–	–	–	–
Watercress	10.90	–	–	–	–	–	0.48	0.13	0.60	0.66	–	–	–	–

1: Ascorbic acid; 2: 3-hydroxybenzoic acid; 3: Syringic acid; 4: 2,5-dihydroxybenzoic acid; 5: 2-hydroxybenzoic acid; 6: Gallic acid; 7: Sinapic acid; 8: *p*-coumaric acid; 9: Ferulic acid; 10: Caffeic acid; 11: Kaempferol; 12: Quercetin; 13: Isovanillic acid; 14: 2,4-dihydroxybenzoic acid; GC-MS: gas cromatography-mass spectrometry.

## Abbreviations

GC-MS: gas chromatography-mass spectrometry
GA: gallic acid
UV: ultraviolet
DPPH^•^: 2,2-diphenyl-1-picryl-hydrazine
ABTS^•+^: 2,2′-azinobis-3-ethylbenzotiazoline-6-sulfonic acid
MSTFA: *N*-methyl-*N*-(trimethylsilyl)trifluoroacetamide
FRAP: ferric reducing antioxidant power
TPC: total phenolic content
Fe^3+^-TPTZ: ferric tripyridyltriazine
SPE: solid phase extraction
TMS: trimethylsilyl
GLM: general linear models
IC_50_: inhibitory concentration
AA: antioxidant activity
DRc: control degradation rat
DRs: degradation rate in the presence of standard or vegetable extract
a: absorbance at initial time (0 min)
b: absorbance at final time (120 min)
BHT: butylated hydroxytoluene
IP: induction period
PF: protection factor
PIa: IP of the oil with the vegetable extracts or standards
Plc: IP of the control (oil without the vegetable extracts or standards)
DW: dry weight
